# Quercitrin protects human bronchial epithelial cells from oxidative damage

**DOI:** 10.1515/med-2022-0416

**Published:** 2022-02-22

**Authors:** Dan Yu, Fan Wang, Shuming Ye, Shuo Yang, Ning Yu, Xinyan Zhou, Nian Zhang

**Affiliations:** Department of Hematology, Wuhan No. 1 Hospital, Wuhan 43022, Hubei, China; General Medical Department (Department of Geriatrics), Wuhan No. 1 Hospital, Wuhan 43022, Hubei, China; Department of Respiratory, Wuhan No. 1 Hospital, Wuhan 43022, Hubei, China; Hubei University of Traditional Chinese Medicine, Wuhan 430061, Hubei, China; Department of Traditional Chinese Medicine, Wuhan No. 1 Hospital, Wuhan 43022, Hubei, China

**Keywords:** chronic obstructive pulmonary disease, quercitrin, oxidative stress, the Nrf2/HO-1/NQO1 pathway, the MAPK/ERK pathway

## Abstract

Chronic obstructive pulmonary disease (COPD) is mainly caused by cigarette smoking (CS), with oxidative stress being one key component during its pathogenesis. This study aimed to investigate the effects of quercitrin (QE) on cigarette smoke extract (CSE)-induced cell apoptosis and oxidative stress in human bronchial epithelial cells (HBECs) and its underlying mechanism. HBECs were treated with 2% CSE for 24 h to establish *in vitro* COPD cellular models. CCK-8 assay and flow cytometry analysis were performed to evaluate cell viability and apoptosis, respectively. Western blotting was applied to examine protein levels and ELISA kits were used to examine contents of the indicated oxidant/antioxidant markers. The results demonstrated that CSE promoted apoptosis and suppressed viability of HBECs and QE reversed these effects. CSE caused increase in T-AOC, superoxide dismutase, and glutathione (GSH) peroxidase contents and decrease in MDA, reactive oxygen species , and GSH contents in HBECs, which were rescued by QE treatment. The CSE-induced Nrf2 nuclear translocation and elevation of NAD(P)H: quinone oxidoreductase 1 (NQO1) and heme oxygenase-1 (HO-1) expression were also reversed by QE in HBECs. The mitogen-activated protein kinase (MAPK) signaling was activated by CSE and further suppressed by QE in HBECs. Collectively, QE exerts a protective role in HBECs against cell apoptosis and oxidative damage via inactivation of the Nrf2/HO-1/NQO1 pathway and the MAPK/ERK pathway.

## 1 Introduction

It has been estimated that chronic obstructive pulmonary disease (COPD) ranks third among leading causes of mortality by 2020 [1]. COPD is characterized by chronic airway inflammation and progressively irreversible airflow limitation [2]. Cigarette smoking (CS) is considered as the predominant factor, accounting for approximately 80–90% of all COPD cases [3]. Oxidative stress is one key component in COPD pathogenesis [4]. Oxidative stress refers to the imbalance of oxidation and antioxidation in the body attacked by harmful stimulating factors [5]. It can directly damage lung tissues and cause gene expression of proinflammatory mediator, exudation of inflammatory cells, and oxidative inactivation of protease, thereby facilitating the development of COPD [6]. Superoxide dismutase (SOD), reactive oxygen species (ROS), glutathione (GSH), and GSH peroxidase (GSH-Px) are markers of oxidative stress [7]. Heme oxygenase-1 (HO-1) is a crucial endogenous enzyme that catalyzes heme decomposition and generates carbon monoxide, biliverdin, and ferrous iron to modulate apoptosis, inflammation, and oxidative stress [8]. NAD(P)H: quinone oxidoreductase 1 (NQO1) is also a significant enzyme related to cell apoptosis and oxidative stress [9,10]. Therefore, exploring the mechanism underlying the oxidative stress in COPD is of great significance for clinical treatment of COPD.

Flavonoids possessing anti-inflammatory and antioxidant activities, easily infiltrate the blood–brain barrier and provide neuroprotection in a series of animal and cellular models of neurological diseases [11,12]. Quercitrin (QE) (2-(3,4-dihydroxyphenyl)-5,7-dihydroxy-4-oxo-4*H*-chromen-3-yl6-deoxyalpha-l-mannopyranoside) is a class of natural flavonoids in the leaves, flowers, and fruits of numerous plants [13,14]. QE was demonstrated to have anti-apoptosis, anti-inflammatory, and anti-oxidative effects [15,16]. Previous reports showed that QE markedly attenuated inflammatory response in the livers of mice as well as reduced acute systemic inflammation in lipopolysaccharide (LPS)-induced models [17]. QE can alleviate apoptosis and inflammation in cytokine-induced models [18]. It has been revealed that QE displays organ protective properties in the kidney, liver, and pancreas by elevating the antioxidant status [19]. Therefore, we aimed to explore the function of QE on apoptosis and oxidative stress in COPD.

Mitogen-activated protein kinase (MAPK) signaling pathway plays a key role in regulating lung inflammation, and can be activated in a cell-stimulus-specific manner [20]. The activation of MAPK signaling pathway involving p38 and ERK has been reported in cigarette smoke extract (CSE)-exposed human bronchial epithelial cells (HBECs) or in COPD patients [21,22]. We intended to explore whether this signaling is involved in cell apoptosis and oxidative stress during COPD progression.

Herein we intended to figure out the specific role of QE in COPD. We established CSE-stimulated cellular models of COPD to elucidate the potential mechanisms of QE on apoptosis and oxidative stress in HBECs, which may provide a potential new direction for COPD treatment.

## 2 Materials and methods

### 2.1 CSE preparation

The smoke from 10 cigarettes was bubbled through 25 mL of media. The suspension was titrated to pH 7.4, filter-sterilized, and regarded as 100% CSE. The CSE sample was diluted with phosphate buffer saline (PBS, Invitrogen, Carlsbad, CA, USA) to gain concentrations of 2%, and CSE was frozen in aliquots at −80°C.

### 2.2 Cell culture and treatment

HBECs were obtained from Chinese Academy of Cell Resource Center (Shanghai, China) and were incubated in Roswell Park Memorial Institute 1640 (RPMI-1640; Sigma-Aldrich, St. Louis, MO, USA) medium containing 10% fetal bovine serum (FBS; Invitrogen) in 5% CO_2_ at 37°C. QE was purchased from Sigma-Aldrich and dissolved in dimethyl sulfoxide (final concentration 0.05%) for cell culture. Cells were treated with QE at different concentrations (0 or 22.4 µg/mL) for 24 h. CSE cellular models were divided into four groups: saline + Con group (0% CSE and 0 µg/mL QE), saline + CSE group (2% CSE and 0 µg/mL QE), QE + Con group (0% CSE and 22.4 µg/mL QE), and QE + CSE group (2% CSE and 22.4 µg/mL QE).

### 2.3 CCK-8 assay

The viability of HBECs was measured by CCK-8 assay. In brief, the cells were dispersed evenly in the medium and then seeded at a density of 4 × 10^4^ cells/well in 96-well plates. Next day, after the cells were treated with indicated reagents, 10 µL of CCK-8 solution (Sigma-Aldrich) was added into each well, followed by 2 h of incubation. The optical density at 450 nm was determined using a microplate reader (Thermo Scientific, Rockford, IL, USA).

### 2.4 Flow cytometry analysis

Flow cytometry was used to evaluate the apoptosis of HBECs. Cells at a density of 5 × 10^4^ cells/well were seeded into 6-well plates, and then harvested and washed. After indicated treatment, cells were resuspended and stained with 5 mL of Annexin V-fluorescein isothiocyanate (FITC) and 1 mL of Propidium Iodide (PI) (Beyotime Biotechnology, Beijing, China) in the dark at room temperature for 10 min. Cell apoptosis rate was analyzed using a FACScan flow cytometry (BD Bioscience, San Jose, CA, USA).

### 2.5 Western blot analysis

Proteins were extracted by RIPA lysis buffer (Beyotime Biotechnology). A nuclear/cytoplasmic isolation kit (Beyotime Biotechnology) was used to obtain cytoplasmic and nuclear extracts. Protein extracts were determined using the BCA assay kit (Sigma-Aldrich). Samples containing equal amount of protein were separated in 10% sodium dodecyl sulfate polyacrylamide gel electrophoresis and transferred onto a nitrocellulose membrane. After blocking with 5% non-fat milk, the membrane was incubated with primary antibodies including anti-Bcl-2 (ab32124, 1:1,000, Abcam, Cambridge, UK), anti-Bax (ab182733, 1:2,000, Abcam), anti-cleaved caspase-3 (ab2302, 1:500, Abcam), anti-HO-1 (ab52947, 1:2,000, Abcam), anti-NQO1 (ab80588, 1:10,000, Abcam), anti-Nrf2 (ab137550, 1:500, Abcam), anti-ERK (ab32537, 1:1,000, Abcam), anti-pERK (ab76299, 1:5,000, Abcam), anti-p38 (ab182453, 1:1,000, Abcam), anti-p-p38 (ab178867, 1:1,000, Abcam), anti-Lamin A/C (ab133256, 1:10,000, Abcam), and anti-β-actin (ab8227, 1:3,000, Abcam) overnight at 4°C. Then, the membranes were incubated with horseradish peroxidase-conjugated secondary antibodies for 2 h at room temperature after being washed with TBST for three times. The signals of protein bands were observed by a chemiluminescence detection system (Thermo Fisher Scientific). Protein expression was normalized to Lamin A/C for nuclear protein and β-actin for total/cytoplasmic protein. The experiment was repeated in triplicate independently.

### 2.6 Measurement of oxidative stress markers

Commercially available ELISA kits (Thermo Scientific) were used to measure total antioxidant capacity (T-AOC), malondialdehyde (MDA), ROS, GSH, SOD, and GSH-Px in HBECs. Results were normalized to protein concentration in HBECs.

### 2.7 Statistical analysis

Data are presented as the mean value ± standard deviation (SD) from at least three independent experiments. Comparisons between two groups or among multiple groups were analyzed by Student’s *t* test or one-way analysis of variance (ANOVA) followed by Tukey’s *post hoc* test using software Prism 7.0 (GraphPad). *P* < 0.05 was considered statistically significant.

## 3 Results

### 3.1 The molecular structure formula of QE

**Figure 1:**
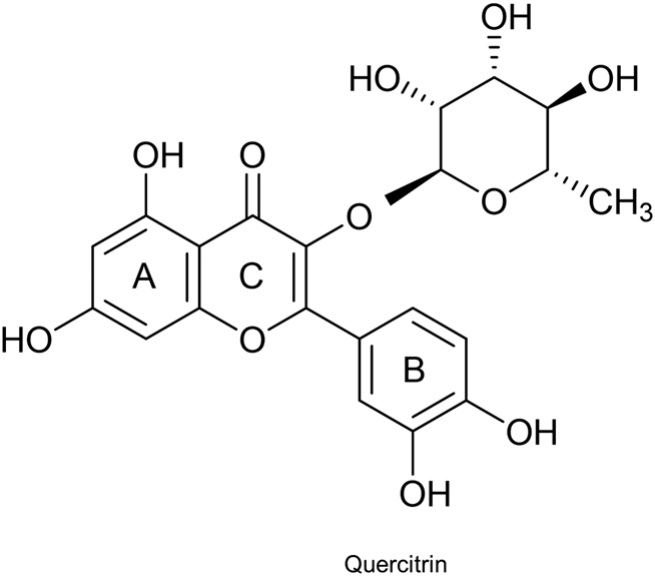
The molecular structure formula of QE.

First, we discovered the molecular structure formula of QE, as shown in Figure 1. QE solubility in polar solvents and its absorption were improved by the sugar portion bound to the aglycone portion [23].

### 3.2 QE rescues the decreased viability and increased apoptosis of CSE-treated HBECs

**Figure 2:**
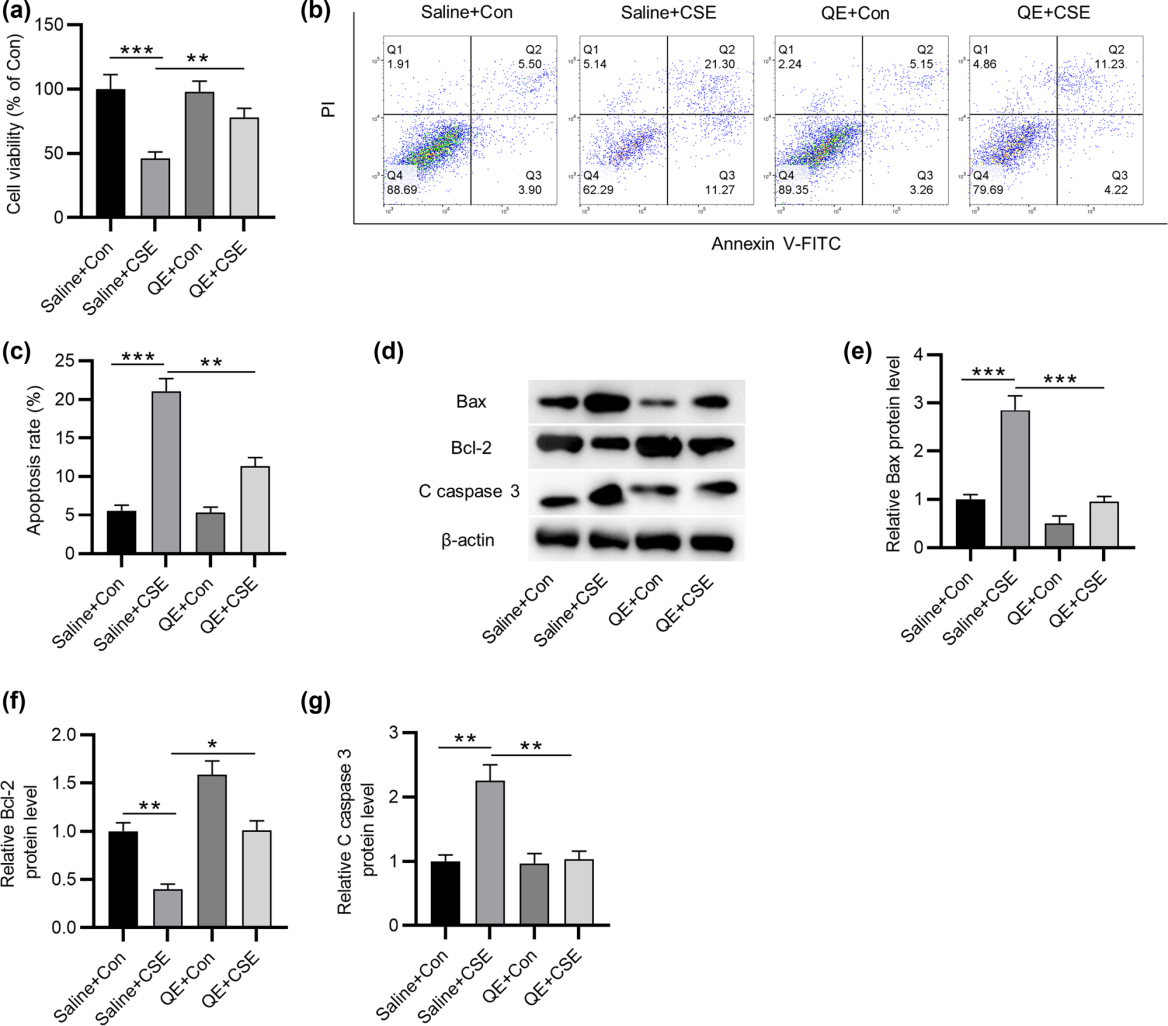
Effects of QE on the viability and apoptosis of CSE-treated HBECs. (a) CCK-8 assay was applied to measure lung epithelial cell viability in saline + Con group, saline + CSE group, QE + Con group, and QE + CSE group. (b and c) Flow cytometry analysis was used to evaluate lung epithelial cell apoptosis in each group. (d–g) Western blotting was performed to detect protein levels of Bax, Bcl-2, and cleaved caspase-3 in HBECs in each group. *p < 0.05, **p < 0.01, and ***p < 0.001.

To explore the biological function of QE in COPD, we mimicked COPD characteristics by establishing CSE-induced cellular models. HBECs were treated with 2% CSE for 24 h. First, CCK-8 assay was performed to evaluate cell viability. The results suggested that CSE treatment attenuated cell viability and QE stimulation partially restored cell viability (Figure 2a). Moreover, flow cytometry analysis suggested that the apoptosis of HBECs was promoted by CES stimulation and rescued by QE treatment (Figure 2b and c). In addition, western blotting depicted that Bax and cleaved caspase-3 protein levels in HBECs were upregulated by CSE stimulation, while Bcl-2 protein level was downregulated after CSE treatment. However, the changes in the protein levels of Bax, Bcl-2, and cleaved caspase-3 caused by CSE stimulation were reversed by QE treatment (Figure 2d–g). Collectively, QE can rescue CSE-induced reduction in lung epithelial cell viability and elevation in cell apoptosis.

### 3.3 QE rescues the CSE-induced oxidant/antioxidant imbalance in HBECs

**Figure 3:**
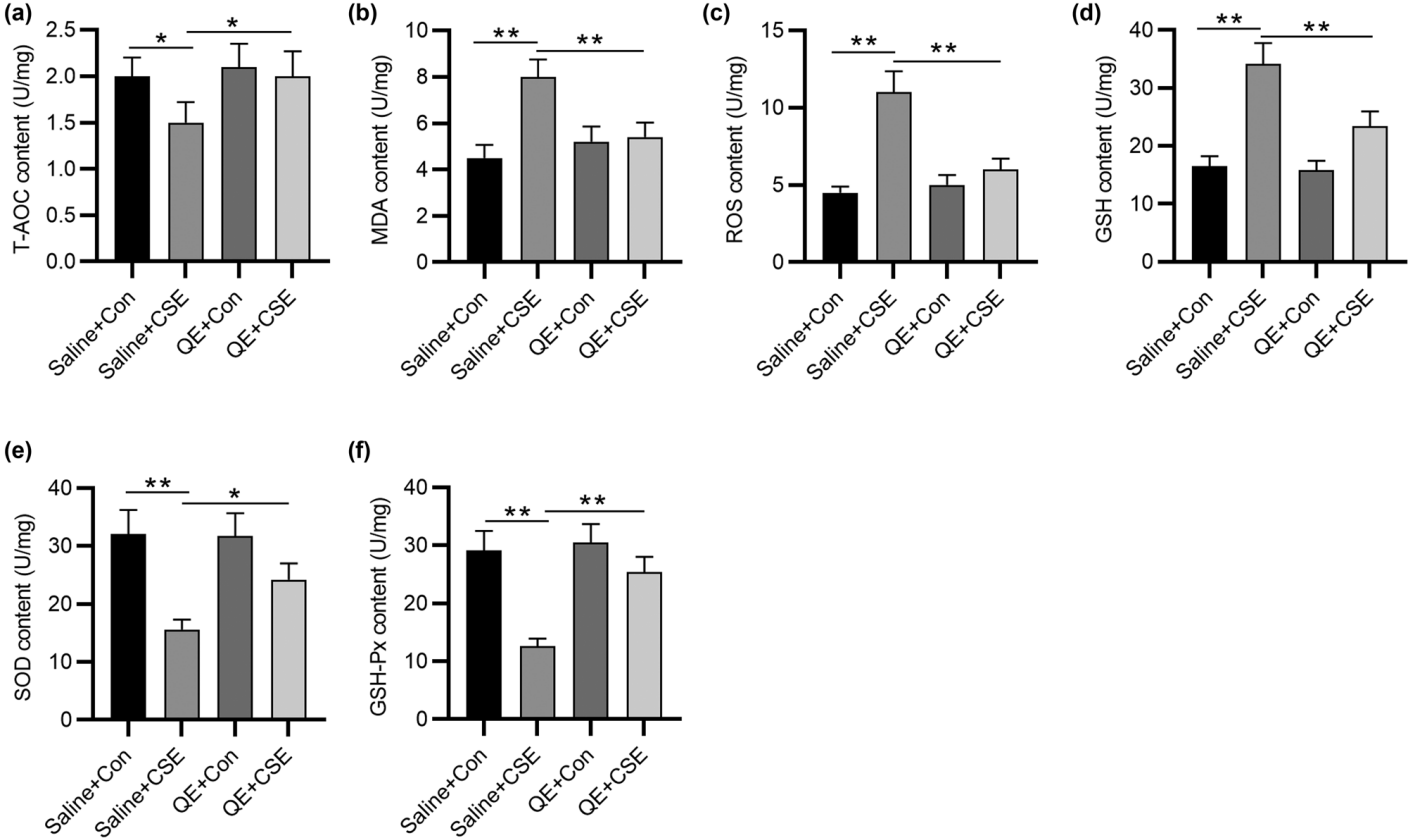
Effects of QE on the contents of oxidative/anti-oxidative markers in CSE-treated HBECs. (a–f) ELISA was used to measure the concentrations of T-AOC, MDA, ROS, GSH, SOD, and GSH-Px in HBECs in saline + Con group, saline + CSE group, QE + Con group, and QE + CSE group. *p < 0.05 and **p < 0.01.

To investigate the anti-oxidative capacity of QE, the content of oxidative/anti-oxidative markers in HBECs were evaluated using ELISA kits. Levels of T-AOC, SOD, and GSH-Px were reduced, while levels of MDA, ROS, and GSH were elevated by CSE exposure (Figure 3a–f). This indicated that CSE led to oxidant damage in HBECs. However, the subsequent QE treatment rescued the elevation of MDA, ROS, and GSH contents as well as reduction in T-AOC, SOD, and GSH-Px contents caused by CSE (Figure 3a–f). Overall, QE alleviates CSE-induced oxidative damage in HBECs.

### 3.4 QE suppresses CSE-induced activation of Nrf2 and antioxidant enzymes HO-1 and NQO-1 in HBECs

**Figure 4:**
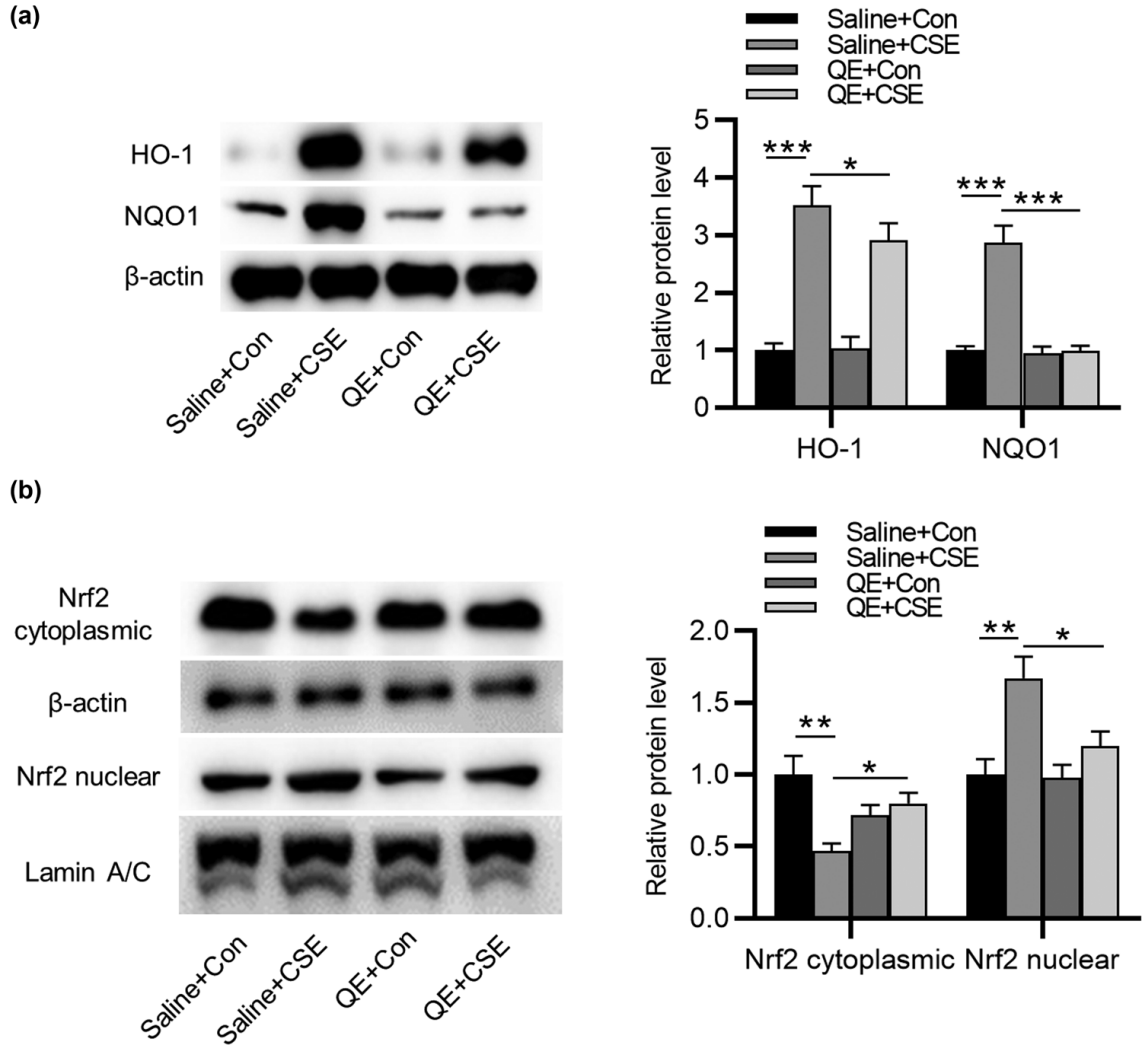
Effects of QE on CSE-induced HO-1, NQO1 enzyme expression, and the Nrf2 translocation in HBECs. (a) Western blotting was used to examine protein levels of NQO1 and HO-1 in HBECs in saline + Con group, saline + CSE group, QE + Con group, and QE + CSE group. (b) Western blotting was used to detect cytoplasmic and nuclear Nrf2 protein expression in HBECs under indicated treatment. *p < 0.05, **p < 0.01, and ***p < 0.001.

Subsequently, we explored the influence of QE on enzyme expression. Through western blotting, we found that CSE treatment markedly upregulated NQO1 and HO-1 protein expression in HBECs compared to saline + Con group, while NQO1 and HO-1 protein levels were significantly decreased in QE + CSE group vs saline + CSE group. QE alone had no significant effect on NQO1 and HO-1 protein level (Figure 4a). Next we evaluated the influence of QE on Nrf2 translocation under CSE treatment since Nrf2 is a key regulator of cytoprotective genes in response to oxidative stress [24]. Nrf2 was observed translocating from cytoplasmic fractions to nuclear fractions in saline + CSE group, while the translocation was rescued by QE treatment in HBECs (Figure 4b). The levels of both cytoplasmic and nuclear Nrf2 showed no significant difference in Saline + Con group and QE + Con group, suggesting that QE alone had no marked effect on CSE-induced Nrf2 translocation. In summary, QE suppresses CSE-induced activation of Nrf2 and antioxidant enzymes HO-1 and NQO-1 in HBECs, thereby alleviating CSE-induced oxidative damage.

### 3.5 QE inactivates the MAPK/ERK signaling pathway in HBECs

**Figure 5:**
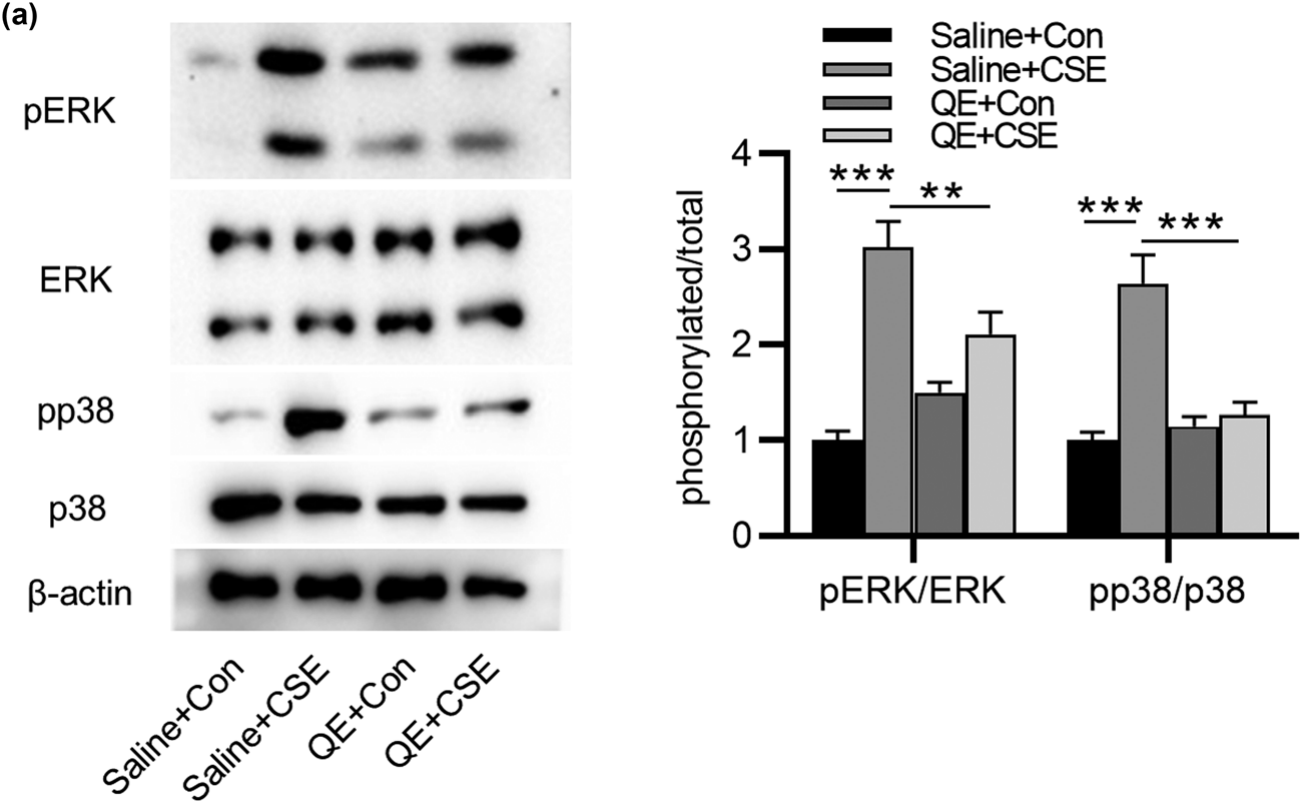
Effects of QE on the MAPK/ERK signaling pathway in CSE-treated HBECs. (a) Western blotting was performed to assess protein levels of ERK, p-ERK, p38, and p-p38 in HBECs in saline + Con group, saline + CSE group, QE + Con group, and QE + CSE group. **p < 0.01 and ***p < 0.001.

We further investigated the underlying mechanism of oxidative stress and apoptosis in HBECs. Previous report has demonstrated that several signaling pathways regulate CSE-induced oxidative stress, involving the MAPK signaling pathway [25]. Western blotting illustrated that the increased phosphorylation of p38 and ERK in HBECs was observed under CSE stimulation in saline + CSE group, which was reversed by QE treatment in QE + CSE group (Figure 5a). Therefore, QE inhibits CSE-induced activation of the MAPK/ERK signaling pathway in HBECs.

## 4 Discussion

In the present study, we demonstrated the protective influence of QE on CSE-induced apoptosis and oxidative stress in HBECs via Nrf2 and MAPK pathways. In our report, we found that CSE promoted apoptosis and induced oxidant/antioxidant imbalance in HBECs, which were rescued by QE treatment. These findings suggested that QE protects HBECs from CSE-induced apoptosis and oxidative damage.

The oxidatant/antioxidant balance depends on the regulation of the endogenous antioxidant defense system [26]. Herein we found that T-AOC, an indicator of total antioxidant status that reflects the degree of oxidative damage in cells [27], was significantly downregulated in CSE-treated HBECs, while antagonized by QE administration. The content of MDA often reflects the degree of lipid peroxidation in tissues and indirectly shows the degree of cell damage [28]. In our report, we discovered a significant increase in MDA content in HBECs under CSE treatment. Nevertheless, as expected, our results indicated that MDA accumulation in HBECs due to CSE was reduced by QE stimulation. Previous reports demonstrated that excessive production of ROS or inadequate anti-oxidative capacity may change the cellular redox balance, thus causing oxidative stress [29]. ROS emitted by cigarette smoke and produced from the structural or inflammatory cells results in direct or indirect damage of lipids, proteins, and nucleic acids, and participate in the pathogenesis of many diseases [30,31,32]. Consistent with this notion, we found that the ROS content in the CSE-treated HBECs was increased; however, the increased ROS was reversed by QE application. In mammalian cells, GSH serves as a main antioxidant that directly or indirectly scavenges free radicals and other reactive nitrogen species via enzymatic reaction [33]. This report revealed that CSE exposure caused a significant increase in GSH concentration, and QE reversed this effect in HBECs. Additionally, SOD enzymes prevent the production of toxic hydroxyl radical via scavenging superoxide anion into hydrogen peroxide and molecular oxygen [34]. In our report, SOD content was reduced by CSE and rescued by QE in HBECs. Furthermore, it has been revealed that GSH-Px is the most abundant and appears to have a major role in ROS defense based on *in vitro* studies [35]. Herein we found that GSH-Px showed decrease under CSE stimulation and further showed reversal under QE treatment in HBECs. These findings suggested that QE alleviates CSE-induced oxidative stress injury in HBECs.

Nrf2 is one of the main cellular defense lines against oxidative stress [36]. Nrf2, as a key transcription factor regulating antioxidant stress, plays an important role in inducing the body’s antioxidant response [37]. Under steady state conditions, unactivated Nrf2 exists in the cytoplasm and is linked to the cytoplasmic protein Keap1 [38]. Keap1 inhibits Nrf2 by retaining Nrf2 in the cytoplasm and enhancing its proteasome degradation through ubiquitination [38]. Under oxidative stress, Nrf2 will be released from the Keap1/Nrf2 complex and transferred to the nucleus, thereby activating Nrf2 and its downstream-regulated genes in the nucleus, including HO-1 and NQO1 [39]. HO-1 is a crucial antioxidative enzyme, which catalyzes the decomposition of heme and generates biliverdin and carbon monoxide, eliminating ROS and maintaining the intracellular redox homeostasis [40]. NQO1 is a quinone oxidoreductase that prevents the production of semiquinone radicals, which are important sources of ROS [41,42]. In animals, Nrf2 activation has been found to attenuate CSE-induced emphysema and airway inflammation in mice [30]. QE was demonstrated to mediate the expression of Nrf2 and the activity of antioxidant response element (ARE)-reporter gene, thus regulating Nrf2/ARE-mediated antioxidant defense mechanism [43]. Previously, in LPS-induced lung injury in mice, the protein level of Cytosol Nrf2 was reduced after LPS treatment, while it was partially restored after GRh2 treatment, which indicated that GRh2 showed antioxidant effects by inhibiting Nrf2 nuclear translocation [44]. In the present study, we discovered that Cytosol Nrf2 was reduced, while Nucleus Nfr2 was elevated after CSE treatment, which suggested that CSE induced oxidative stress and the subsequent Nrf2 nuclear translocation. Nevertheless, QE reversed the effects of CSE on Nrf2. Additionally, the elevation in HO-1 and NQO1 levels caused by CSE was restored by QE treatment. Therefore, QE suppressed CSE-induced oxidative stress, thereby restraining Nrf2 nuclear translocation and activation antioxidant enzymes HO-1 and NQO-1. Furthermore, the MAPK pathway is one of the signaling pathway in lung diseases and is closely associated with oxidative stress [45,46]. QE has been reported to suppress the phosphorylation of p38 MAPK and ERK, and downregulate MAPK signaling pathways before [47,48]. In our research, the protein level of p-ERK and p-p38 was elevated by CSE and attenuated by QE, which indicated the suppressive function of QE on activation of the MAPK pathway. These findings suggested that QE exerts a protective role against oxidative damage and apoptosis in HBECs through the inactivation of the Nrf2 and MAPK pathways.

There also exist some limitations in this study. First, the function of QE on cell apoptosis and oxidative stress in COPD was evaluated only by treating HBECs with CSE to establish *in vitro* cellular model. The conduction of *in vivo* experiments using animal models of COPD will make our results more convincing. Second, chronic inflammation is also a crucial factor attributing to the pathogenesis of COPD. QE extracted from Houttuynia cordata used for treating inflammation-related disorders was reported to markedly repress lung inflammatory response in the mouse model of LPS-induced acute lung injury [49]. However, our study only investigated and proved that QE can inhibit CSE-induced cell apoptosis and oxidative stress in HBECs. Therefore, whether QE protects HBECs from CSE-induced inflammation required further exploration in future studies.

In conclusion, our research innovatively put forward and confirmed that QE can reverse CSE-induced oxidative stress and apoptosis in HBECs by inactivating the Nrf2 and MAPK signaling pathways. This report suggested that QE has the potential to be developed as an effective agent for improving clinical treatment of COPD.
